# Integrated Proteo-Transcriptomic Analyses Reveal Insights into Regulation of Pollen Development Stages and Dynamics of Cellular Response to Apple Fruit Crinkle Viroid (AFCVd)-Infection in *Nicotiana tabacum*

**DOI:** 10.3390/ijms21228700

**Published:** 2020-11-18

**Authors:** Ankita Shrestha, Ajay Kumar Mishra, Jaroslav Matoušek, Lenka Steinbachová, David Potěšil, Vishnu Sukumari Nath, Praveen Awasthi, Tomáš Kocábek, Jernej Jakse, Lenka Záveská Drábková, Zbyněk Zdráhal, David Honys, Gerhard Steger

**Affiliations:** 1 Biology Centre, Czech Academy of Sciences, Department of Molecular Genetics, Institute of Plant Molecular Biology, Branišovská 31, 37005 České Budějovice, Czech Republic; ankita.shrestha@umbr.cas.cz (A.S.); jmat@umbr.cas.cz (J.M.); sukumari.nath@umbr.cas.cz (V.S.N.); praveen.awasthi@umbr.cas.cz (P.A.); kocabek@umbr.cas.cz (T.K.); 2 Institute of Experimental Botany of the Czech Academy of Sciences, Rozvojová 263, 165 02 Prague 6-Lysolaje, Czech Republic; steinbachova@ueb.cas.cz (L.S.); l.zaveska.drabkova@ueb.cas.cz (L.Z.D.); honys@ueb.cas.cz (D.H.); 3 Mendel Centre for Plant Genomics and Proteomics, Central European Institute of Technology, Masaryk University, Kamenice 5, 625 00 Brno, Czech Republic; dpotesil@mail.muni.cz (D.P.); zbynek.zdrahal@ceitec.muni.cz (Z.Z.); 4 Department of Agronomy, Biotechnical Faculty, University of Ljubljana, Jamnikarjeva 101, SI-1000 Ljubljana, Slovenia; Jernej.Jakse@bf.uni-lj.si; 5 Institut für Physikalische Biologie, Heinrich-Heine-Universität Düsseldorf, D-40204 Düsseldorf, Germany; steger@biophys.uni-duesseldorf.de

**Keywords:** AFCVd propagation and eradication, viroid replication, viroid degradation, *Nicotiana tabacum*, male gametophyte, Proteome, RNA sequencing, RT qPCR

## Abstract

Tobacco (*Nicotiana tabacum*) pollen is a well-suited model for studying many fundamental biological processes owing to its well-defined and distinct development stages. It is also one of the major agents involved in the transmission of infectious viroids, which is the primary mechanism of viroid pathogenicity in plants. However, some viroids are non-transmissible and may be possibly degraded or eliminated during the gradual process of pollen development maturation. The molecular details behind the response of developing pollen against the apple fruit crinkle viroid (AFCVd) infection and viroid eradication is largely unknown. In this study, we performed an integrative analysis of the transcriptome and proteome profiles to disentangle the molecular cascade of events governing the three pollen development stages: early bicellular pollen (stage 3, S3), late bicellular pollen (stage 5, S5), and 6 h-pollen tube (PT6). The integrated analysis delivered the molecular portraits of the developing pollen against AFCVd infection, including mechanistic insights into the viroid eradication during the last steps of pollen development. The isobaric tags for label-free relative quantification (iTRAQ) with digital gene expression (DGE) experiments led us to reliably identify subsets of 5321, 5286, and 6923 proteins and 64,033, 60,597, and 46,640 expressed genes in S3, S5, and PT6, respectively. In these subsets, 2234, 2108 proteins and 9207 and 14,065 mRNAs were differentially expressed in pairwise comparisons of three stages S5 vs. S3 and PT6 vs. S5 of control pollen in tobacco. Correlation analysis between the abundance of differentially expressed mRNAs (DEGs) and differentially expressed proteins (DEPs) in pairwise comparisons of three stages of pollen revealed numerous discordant changes in mRNA/protein pairs. Only a modest correlation was observed, indicative of divergent transcription, and its regulation and importance of post-transcriptional events in the determination of the fate of early and late pollen development in tobacco. The functional and enrichment analysis of correlated DEGs/DEPs revealed the activation in pathways involved in carbohydrate metabolism, amino acid metabolism, lipid metabolism, and cofactor as well as vitamin metabolism, which points to the importance of these metabolic pathways in pollen development. Furthermore, the detailed picture of AFCVd-infected correlated DEGs/DEPs was obtained in pairwise comparisons of three stages of infected pollen. The AFCVd infection caused the modulation of several genes involved in protein degradation, nuclear transport, phytohormone signaling, defense response, and phosphorylation. Intriguingly, we also identified several factors including, DNA-dependent RNA-polymerase, ribosomal protein, Argonaute (AGO) proteins, nucleotide binding proteins, and RNA exonucleases, which may plausibly involve in viroid stabilization and eradication during the last steps of pollen development. The present study provides essential insights into the transcriptional and translational dynamics of tobacco pollen, which further strengthens our understanding of plant-viroid interactions and support for future mechanistic studies directed at delineating the functional role of candidate factors involved in viroid elimination.

## 1. Introduction

Viroids encompass a group of small (246–401 nucleotides long) circular single-stranded RNA molecules with independently replicating genomes [1]. They are known to be infectious and etiologic agents causing severe economic losses in crops, vegetables, fruits, as well as ornamental plants [2]. Viroids lack their own translational machinery and, hence, depend on the host transcriptional machinery for carrying out replication [3]. Viroids are classified into two families based on their mode and site of replication, the presence/absence of a hammerhead ribozyme, and structural properties—members of *Pospiviroidae*, which replicate in the nucleus, and *Avsunviroidae*, which replicate (and accumulate) in the chloroplast [4]. Members of the Avsunviroidae family, such as avocado sunblotch viroid (ASBVd) and peach latent mosaic viroid (PLMVd), require the nuclear-encoded chloroplastic DNA-dependent RNA polymerase, which transcribes (+) as well (−) strands into oligomeric intermediates that are cleaved into monomers by viroid-encoded hammerhead ribozymes [5]. Members of Pospiviroidae essentially require the DNA-dependent RNA polymerase II (Pol II) for transcription, which transcribe (+) polarity strands into oligomeric (−) RNAs, which serve as a template for the production of linear oligomeric positive ssRNA [6]. Intriguingly, some previous reports have shown that viroids can directly interact with host protein for their replication (such as ribosomal protein L5, TF IIIA (TFIIIA-9ZF) and its variant (TFIIIA-7ZF) [7], and systemic spread using host proteins, such as phloem RNA binding protein PP2 and Virp1 [8,9]. Over the past years several studies have focused on unraveling the mechanism of viroid pathogenesis by RNA-mediated gene silencing, which suggested that viroid-derived small RNA (vd-sRNAs) are involved in post transcriptional gene silencing (PTGS) of host mRNAs leading to symptom development [10]. In addition to activation of PTGS, vd-sRNAs of Pospiviroidae members can be loaded onto an Argonaute protein (AGO4) and direct the DNA methylation on homologous DNA sequences, or modify DNA methylation of ribosomal RNA (rRNA) genes based on an RNA-dependent DNA methylation (RdDM) mechanism, together with other components of the transcriptional gene silencing (TGS) machinery [11].

The transmissibility of a viroid and its ability to infect a specific plant host range is an important and intriguing phenomenon. Viroids majorly employ the horizontal mode of transmission for their dispersal, which includes mechanical transmission by infected farming equipment and pollination by insects [12]. The vertical transmission of viroids involves the dissemination of the infectious agent via infected pollen or seeds [13,14]. Pollen-transmission of viroids is an important pathway to spread their way through progenies (seeds), depending on the viroid and plant species, viroid structure, infection stage, and environmental conditions [15]. Pollen-transmissibility has been reported for several viroids, such as hop stunt viroid (HSVd), ASBVd, and chrysanthemum stunt viroid (CSVd). Interestingly, some viroid species, such as apple scar skin viroid (ASSVd), dapple apple viroid (DAVd), and apple fruit crinkle viroid (AFCVd), are detected in somatic anther tissue, but not detected in mature pollen of infected plants. Consequently, they cannot be transmitted, either by vertical or horizontal transmission to their corresponding host [15,16,17]. This indicates that some viroids are non-transmissible via pollen due to their elimination or suppression during the process of pollen maturation/germination. Our recent experimental evidences indicated that intimate molecular interaction(s) of AFCVd, and its recognition and elimination on the level of male gametophyte in tobacco occurs due to depression of viroid replication machinery, and increase in viroid degradation, which cause the complete degradation of circular and linear viroid forms [17].

The tobacco (*Nicotiana tabacum*) pollen or male gametophyte formation occurs via two distinct and successive developmental phases, microsporogenesis, and microgametogenesis [18]. During microsporogenesis, microsporocytes undergo meiosis, followed by cytokinesis to generate a tetrad of four haploid microspores, whereas microgametogenesis is marked by two stereotypical mitotic divisions. The first mitotic divisions give rise to early bicellular pollen (stage 3, S3), which subsequently grows and matures as late bicellular pollen (stage 5, S5) before it finally desiccates, with second mitosis, which occurs during pollen tube growth within the pistil [19]. Pollen development consists of a series of complex events, which offers an interesting realm to analyze the stage-specific gene expression patterns and their regulation at transcriptome and proteome level. The pollen development has been extensively studied in Arabidopsis and rice, and expressed genes were shown to be enriched for signaling, cell cycle regulation, metabolism, transport, transcription and translation [20,21]. However, limited work has been dedicated to studying changes that occur in the transcriptomes at different stages of tobacco [22,23,24]. The transcriptome landscape is a moderate predictor for protein expression without accounting for post-transcriptional regulation, which has been shown to play a crucial role in modulating gene expression in spatial and temporal manner. Considering this lacuna, we performed a combination of high throughput transcriptome and proteome profiling for three developmental pollen stages, namely early bicellular pollen (S3), late bicellular pollen (S5), and 6 h-pollen tube (PT6) to gain insight into gene expression, and the importance of translational regulation of protein abundance at the individual stages of pollen development.

Along with unraveling the mechanisms of viroid pathogenicity in plants, we recently described the decrease in viroid replication and elimination during S3 to S5 to mature pollen grain and the involvement of putative factors responsible for these events [17]. To get a comprehensive view on responses of the three developmental stages to the AFCVd-infection process and mechanism that governs the molecular/cellular processes of viroid degradation during pollen development, we integrated high-throughput transcriptome and proteome profiling of S3, S5, and the PT6 for both healthy and AFCVd-infected tobacco. The combined approaches and simultaneous analysis of the three developmental stages with and without AFCVd-infection allowed us to capture significant metabolic rearrangements occurring as a result of stage transition and infection with AFCVd. We assume that this study in continuation with our previous findings will lead to a better understanding of the molecular mechanism of viroid protection and degradation that simultaneously occurs in the pollen tissue leading to decrease in viroid propagation.

## 2. Results

### 2.1. Transcriptome Sequencing and Assembly

For a better understanding of the preferential expression of genes during the three developmental stages of control (CT) male gametophyte (CT_S3, CT_S5 and CT_PT6), and transcriptional events in response to AFCVd-infection during the same three developmental stages (AI_S3, AI_S5 and AI_PT6), we constructed RNA libraries from each control and infection condition. Sequencing of the libraries in duplicates resulted in a minimum of 33 million and maximum of 115 million reads. The average number of reads had a median quality scores were above 30, suggesting high quality sequences. An overview of the statistics on transcriptome sequencing and assembly of three stages of male gametophyte development with or without AFCVd-infection is presented in Table 1.

**Figure 1:**
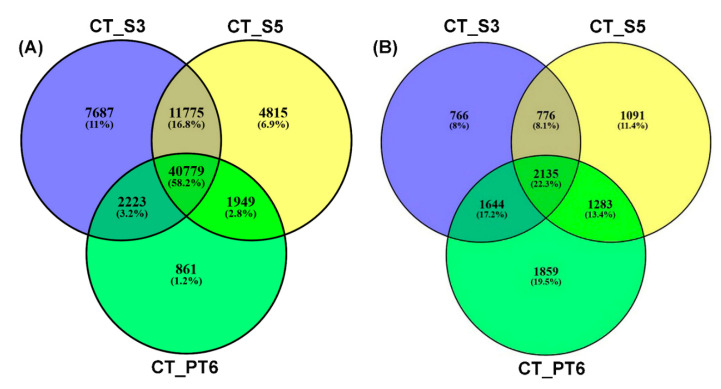
Venn diagram of transcriptome and proteome. Number of transcripts (**A**) and proteins (**B**) identified in stage 3 (CT_S3), stage 5 (CT_S5), and pollen tube after 6 hours of germination (CT_PT6) in control samples.

The transcriptome assemblies based on the reference-genome after mapping filtered reads to the *N. tabacum* reference genome predicted 64,033, 60,597, and 46,640 genes in the CT_S3, CT_S5, and CT_PT6 stages, respectively, suggesting the diminution of the transcriptome during the progression of male gametophyte development. Among them, 40,779 genes were expressed in all three stages (Figure 1A).

The principal component analysis (PCA) obtained from transcriptome replicate dataset exhibited variances between control and AI plants that were low for the same but high among the different developmental stages (Figure S1A). The expression profiles displayed considerable differences in gene expression patterns in the three stages of male gametophyte development. A total of 9808 and 14,065 genes were differentially expressed between CT_S5 vs. CT_S3, and CT_PT6 vs. CT_S5, respectively (Tables S1 and S2), of which, 4613 were shared by two pairs in pairwise stage comparison (Figure S2(AI)). The total number of downregulated differentially expressed mRNAs (DEGs) was higher than the number of upregulated genes across the male gametophyte developmental stages. In addition, analysis of the mapped reads showed that the AFCVd infection caused a significant differential expression of 4766 and 5840 tobacco transcripts in late bicellular pollen and 6h-pollen tube, respectively (Figure S2(AII)), with a larger number of suppressed transcripts (Tables S3 and S4), among which the majority of developmental stage-specific DEGs were further modulated due to AFCVd-infection (Figure S2(AIII,AIV)).

### 2.2. Overview of Quantitative Proteomics Analysis

The PCA of the LC-MS/MS libraries obtained from the three developmental stages of both CT- and AI male gametophytes, showed that the biological replicates were well correlated with observed variance across all developmental stages, and between individual control and corresponding AI stages (Figure S1B). Approximately, 84.2−86.8% of the proteins were quantified in all three biological replicates, while 8.3−11.21% of identified proteins were present in at least two biological replicates, demonstrating the high reproducibility of proteome coverage across the biological replicates (Table S5). The number of identified proteins was 5321, 5286, and 6921 in the three corresponding developmental stages (Table 2).

Of the 12,063 identified proteins, 2135 were present in all three developmental stages of male gametophyte (Figure 1B). The statistical analysis identified 2234 and 2107 proteins as differentially expressed proteins (DEPs) in one to one comparison between CT_S5 vs. CT_S3 and CT_PT6 vs. CT_S5, respectively (Tables S1 and S2). Among the identified DEPs, 390 existed in both CT_S5 vs. CT_S3 and CT_PT6 vs. CT_S5 stages comparisons (Figure S2(BI)). Similarly, a total of 2224 and 1309 different proteins were consistently identified and quantified, which were affected in AI_S5 vs. AI_S3 and AI_PT6 vs. AI_S5, respectively (Tables S3 and S4). A total number of 215 proteins were identified as DEPs in pairwise comparison between AI_S5 vs. AI_S3 and AI_PT6 vs. AI_S5 (Figure S2(BII)). In addition, some of the developmental stage-specific DEPs were further modulated due to AFCVd-infection (Figure S2(BIII,BIV)).

### 2.3. Correlation of Transcript and Protein Profiles

In order to integrate our proteomic data with the transcriptomic data, the dynamic range of the expression pattern of all identified proteins and their corresponding transcript was investigated within a single developmental stage as well as between the three different developmental stages with and without AFCVd-infection (Figure S2(CI–IV)). Strikingly, in the comparison between three consecutive developmental stages, the one-to-one link between the number of expressed transcripts and number of detected proteins were 4.0%, 4.2%, and 6.8% in CT_S3, CT_S5, and CT-PT6 datasets, respectively (Table 2). However, the Pearson’s correlation coefficients of all quantitative DEPs and their corresponding DEGs, in one-to-one comparison between CT_S5 vs. CT_S3, CT_PT6 vs. CT_S5, AI_S5 vs. AI_S3, and AI_PT6 vs. AI_S5 were 0.02, 0.03, 0.1, and 0.27, respectively (Table 2), suggesting that the relationship between mRNA and protein levels was modest between transcriptome and proteome datasets. Notably, a similar expression profile of the transcripts linked to proteins was established for only 4.8% and 3.7% of the identified proteins, while 28.19% and 35.71% of them followed the opposite trend of protein expression based on their protein-to-transcript ratio (PTR values) [25] in CT and AI datasets, respectively.

### 2.4. Clustering, Functional Annotation, and Enrichment Analysis of Correlated DEGS and DEPs

**Figure 2:**
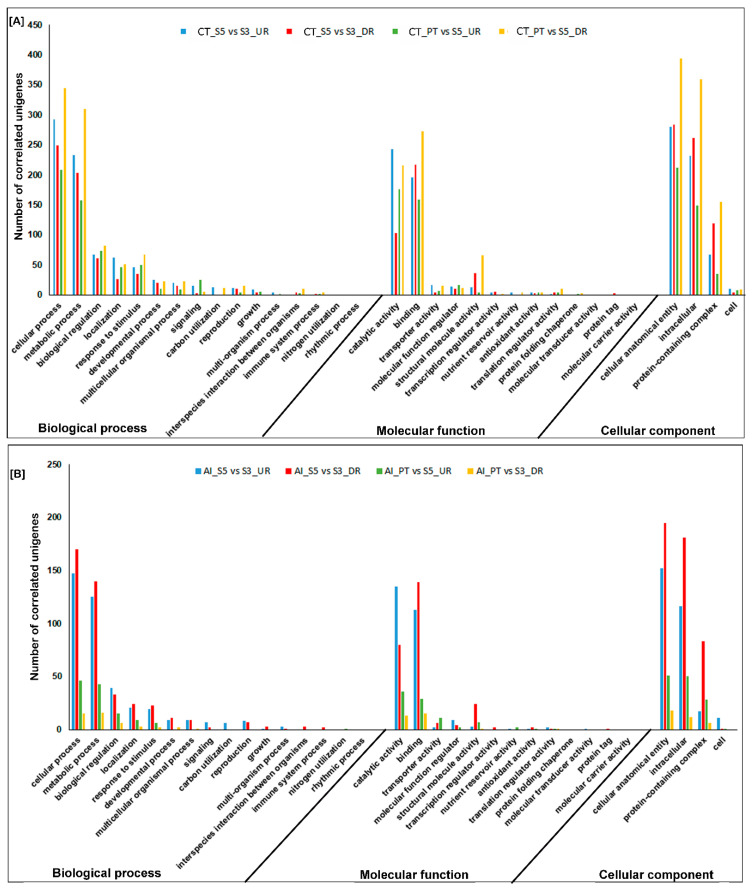
Histogram of Gene Ontology (GO) classification of correlated differentially expressed mRNAs/differentially expressed proteins (DEGs/DEPs) in stage-specific comparison in control (**A**) and AFCVd-infected (**B**) pollen. The results are summarized in three main categories: biological process, cellular component, and molecular function. The *x*-axis indicates subcategories; right *y*-axis indicates number of genes in a category. UR: upregulated, DR: downregulated.

To gain insights into the functional differences between the three developmental stages of male gametophyte and their biological response against AFCVd-infection, Gene Ontology (GO) terms were extracted for correlated DEGs and DEPs (cor-DEGs/DEPs) and subjected to GO enrichment analysis. A total of 3321 and 3636 GO terms were mapped against cor-DEGs/DEPs in stagewise comparison between CT_S5 vs. CT_S3, CT_PT6 vs. CT_S5, respectively. The cor-DEGs/DEPs of AI stages in two group comparisons, AI_S5 vs. AI_S3 and AI_PT6 vs. AI_S5, when mapped against the same GO categories, resulted in identification 2332 and 505 GO terms, respectively. In the two-stage pairwise comparison of CT groups, the differences were mainly reflected in “response to stimulus” and “developmental process” of biological process, and “structural molecule activity” of molecular function (Figure 2A), suggesting that these functional categories might play crucial roles in pollen development. The biological processes categories “cellular process”, “metabolic process” and “biological regulation” molecular function categories “catalytic activity” and “binding” were dominantly represented in AI two-stage comparison (Figure 2B).

**Figure 3:**
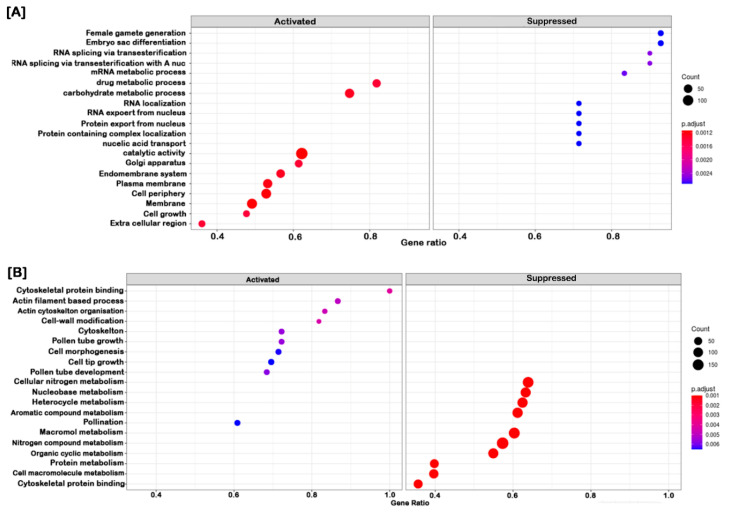
GO functional enrichment analysis of correlated DEGs/DEPs in pairwise comparison of S5 vs. S3 (**A**) and PT6 vs. S5 (**B**) of control male gametophyte of tobacco. The *y*-axis indicates gene functions, and the *x*-axis indicates gene ratios. The size of the dots represents the degree of enrichment and their color indicates degree of statistical significance.

**Figure 4:**
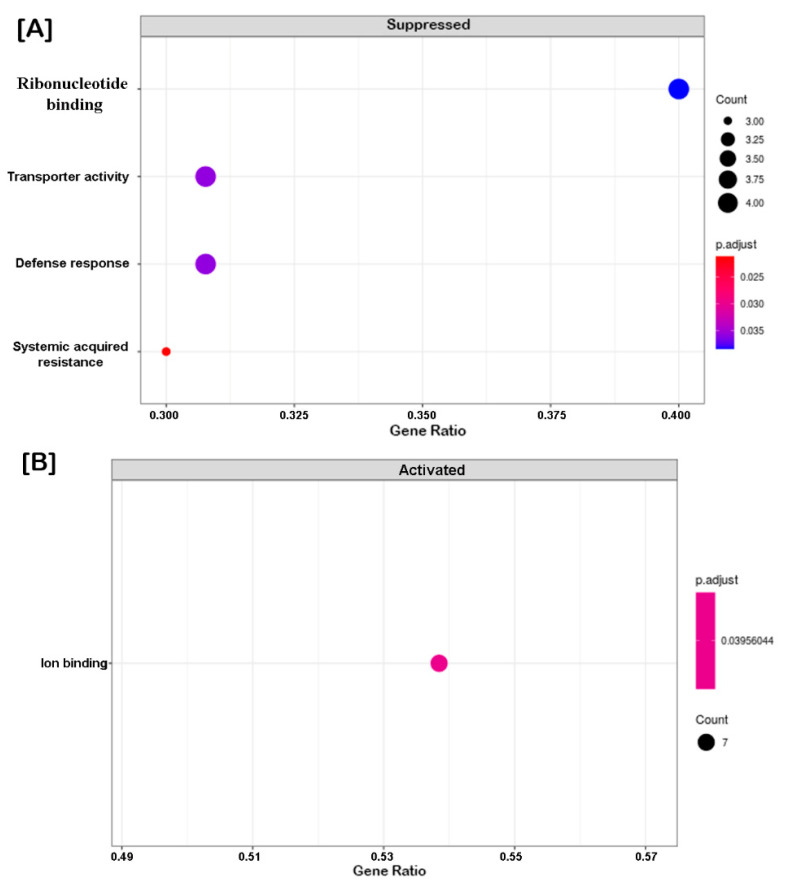
GO functional enrichment analysis of correlated DEGs/DEPs in pairwise comparisons of S5 vs. S3 (**A**) and PT6 vs. S5 (**B**) of AFCVd-infected male gametophyte of tobacco. The *y*-axis indicates gene functions, and the *x*-axis indicates gene ratios. The size of dots represents the degree of enrichment and their color indicates the degree of statistical significance.

Furthermore, the GO enrichment analysis provided the statistically significant top 20 GO terms of cor-DEGs/DEPs in developmental changes (Figure 3) and differential responses to AFCVd-infection (Figure 4) at system-level functional pathways. In the comparison between CT_S5 vs. CT_S3, we observed an enrichment in GO terms of upregulated cor-DEGs/DEPs related to “carbohydrate metabolic process” and “catalytic activity”, etc. (Figure 3A), whereas in CT_PT6 vs. CT_S5, the most significantly enriched terms were “actin filament-based process”, “cell morphogenesis”, “pollen tube growth” and “pollen tube development”, etc. (Figure 3B). In contrast, when the pollen were infected with AFCVd, the more representative enrichment of cor-DEGs/DEPs was related to “transporter activity”, “defense response” and “systemic acquired resistance” in AI_S5 vs. AI_S3 (Figure 4A), while only “ion binding” was observed in AI_PT6 vs. AI_S5 (Figure 4B). Comparisons of CT_S5 vs. CT_S3, and AI_S5 vs. AI_S3 revealed some interesting trends of downregulated cor-DEGs/DEPs in pathways related to “proteins import into nucleus”, “nucleotide binding” and “ribonucleotide binding”, suggesting the impairment of AFCVd replication and propagation during S3 to S5 transition.

To further dissect the biological processes and pathway assignments, where these cor-DEGs/DEPs are involved, pathway-based analysis was performed based on Kyoto Encyclopedia of Genes and Genomes (KEGG) database. The results showed that a total of 567, 583, 485, and 92 cor-DEGs/DEPs from the CT_S5 vs. CT_S3, CT_PT6 vs. CT_S5, AI_S5 vs. AI_S3, and AI_PT6 vs. AI_S5, respectively, were assigned to 234, 274, 186, and 98 KEGG pathways. According to the KEGG annotation, cor-DEGs/DEPs related to carbohydrate metabolism, energy metabolism, translation, folding, sorting and degradation, and signal transduction were predominantly differentially expressed in one-to-one comparison in CT_PT6 vs. CT_S5, AI_PT6 vs. AI_S5 compared to CT_S5 vs. CT_S3 and AI_S5 vs. AI_S3, respectively (Table 3).

Significantly enriched pathway terms were determined for CT_S5 vs. CT_S3 and CT_PT6 vs. CT_S5 stage comparisons based on standard *p*-value <  0.05 and corrected *p*-value according to the number of cor-DEGs/DEPs in each term of the KEGG pathway in comparison with the background number. The results showed that the three KEGG pathways, namely ’’glycolysis”, “metabolic pathway” and “biosynthesis of secondary metabolite”, were highly enriched at both mRNA and protein levels in CT_S5 vs. CT_S3 (Figure S3A), while “phenylpropanoid biosynthesis”, “pentose and glucuronate interconversions” and “endocytosis” were significantly enriched in both the proteome and transcriptome for the cor-DEGs/DEPs in CT_PT6 vs. CT_S5 (Figure S3B). Furthermore, the KEGG enrichment analysis revealed that RNA degradation process was disrupted and ribosome biogenesis was enriched in AI_S5 vs. AI_S3 (Figure S3C) and AI_PT6 vs. AI_S5 (Figure S3D), respectively, at both protein and transcriptome levels in response to AFCVd-infection.

**Figure 5:**
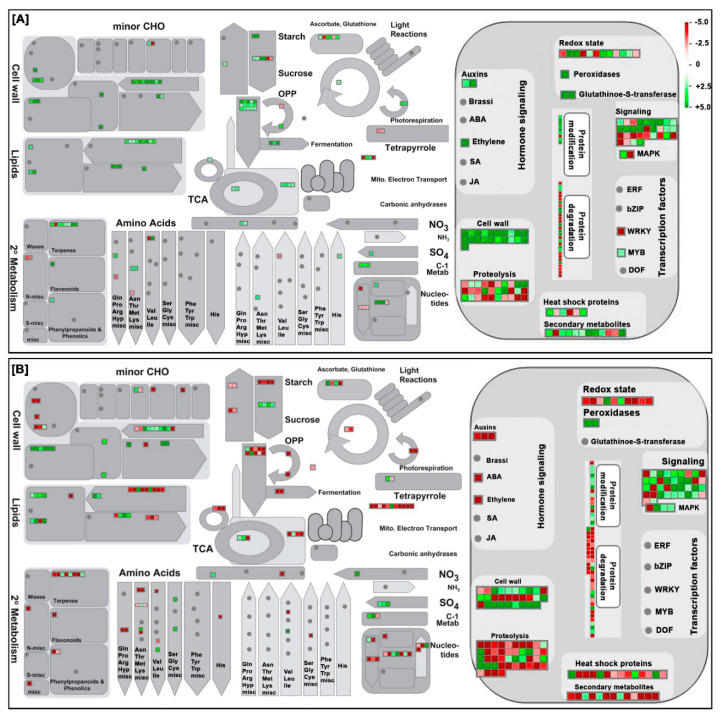
Overview of the MapMan visualization of correlated DEGs/DEPs in pairwise comparisons of S5 vs. S3 (**A**) and PT6 vs. S5 (**B**) of healthy (control) male gametophyte of tobacco. The log2 fold changes of significantly modulated cor-DEGs/DEPs were imported and visualized in MapMan. Signals colored red and green represent a decrease or increase in transcript abundance in the pairwise comparison sets. The scale used for coloration of the signals (log2 ratios) is shown on the top right.

**Figure 6:**
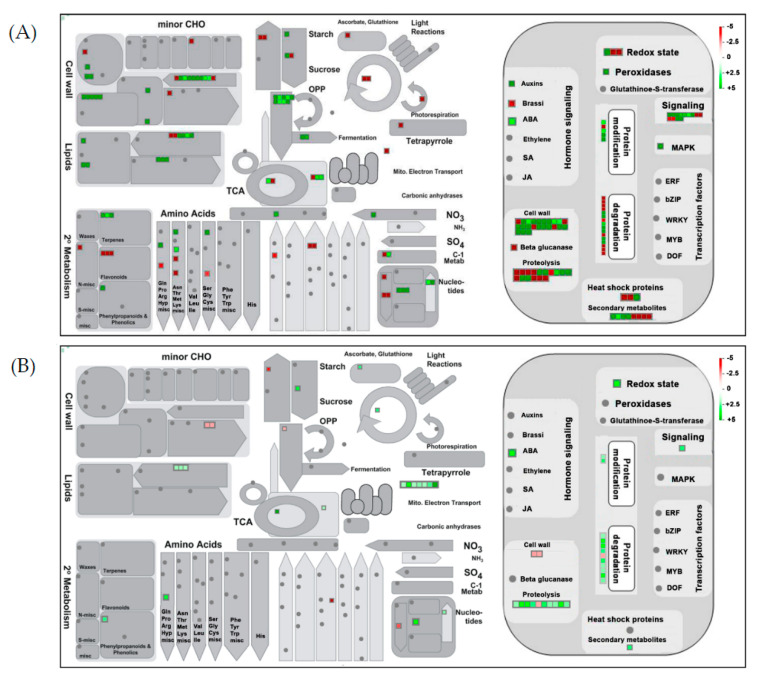
Overview of the MapMan visualization of correlated DEGs/DEPs in pairwise comparison of S5 vs. S3 (**A**) and PT6 vs. S5 (**B**) of AFCVd-infected male gametophyte of tobacco. The log2 fold changes of significantly cor-DEGs/DEPs were imported and visualized in MapMan. Signals colored red and green represent a decrease or increase in transcript abundance in the pairwise comparison sets of three AFCVd-infected stages. The scale used for coloration of the signals (log2 ratios) is shown on the top right.

To gain an unbiased and systematic overview of cor-DEGs/DEPs in the regulation of biological processes and cellular functions during AFCVd-infection and in the three distinct developmental stages, we performed the MapMan pathway-based analysis. In the two comparisons, CT_S5 vs. CT_S3 showed a significant upregulation in cell wall biosynthesis, shikimate pathway, CHO metabolism, secondary metabolism, hormone signaling and ubiquitination (Figure 5A), while in CT_PT6 vs. CT_S5, all categories were approximately divided equally between up- and downregulation (Figure 5B). Upon focusing on biotic stresses, the major difference in expression trends was observed in proteolysis, chaperone activity, oxidative pentose phosphate, tricarboxylic acid cycle, depicting the intricate nexus of molecular changes induced in AI_S5 vs. AI_S3 (Figure 6A) and AI_PT6 vs. AI_S5 (Figure 6B).

**Figure 7:**
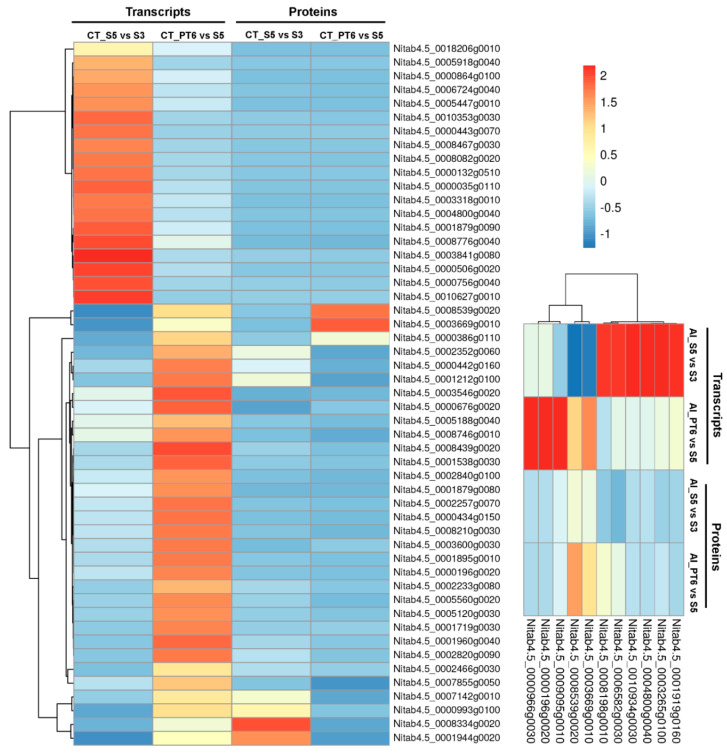
Cluster analysis of associated correlated DEGs/DEPs at transcript and protein levels in pairwise comparison of control and AFCVd (AI) samples of S3, S5, and PT6. Each row in the graph represents a protein/mRNA, and each column in the graph represents the log_2_ transformed average FPKM value of DEGs of two biological replicates (n = 2) and log_2_ transformed average iTRAQ value of corresponding DEPs of three biological replicates (n = 3). Expression differences are shown in different colors; red indicates up-regulation, while blue indicates down-regulation. The color scale of the heat map ranges from saturated blue (value, −2.0) to saturated red (value, 2.0) in the natural logarithmic scale.

Among the common 51 cor-DEGs/DEPs in CT_S5 vs. CT_S3, and CT_PT6 vs. CT_S5 group comparisons, 41 genes displayed the similar expression trend, while 10 genes displayed the opposite trend between the proteomic and transcriptomic data (Figure 7). Similarly, between the AI_S5 vs. AI_S3, and AI_PT6 vs. AI_S5 group comparison, 29 of the 51 genes displayed the similar trend, while 22 displayed the opposite expression trend at the proteomic and transcriptomic levels (Figure 7). These clusters clearly represented different gene expression patterns during development and response against AFCVd-infection.

### 2.5. Validation of Gene Expression Levels

Real-time quantitative PCR (qRT-PCR) experiment was performed to validate the RNA-seq results and investigate the interrelationship between mRNA abundance and protein levels. The transcripts of the selected genes (cor-DEGs/DEPs) involved in pollen development displayed different expression profiles in the two comparison groups of three developmental stages of male gametophyte.

**Figure 8:**
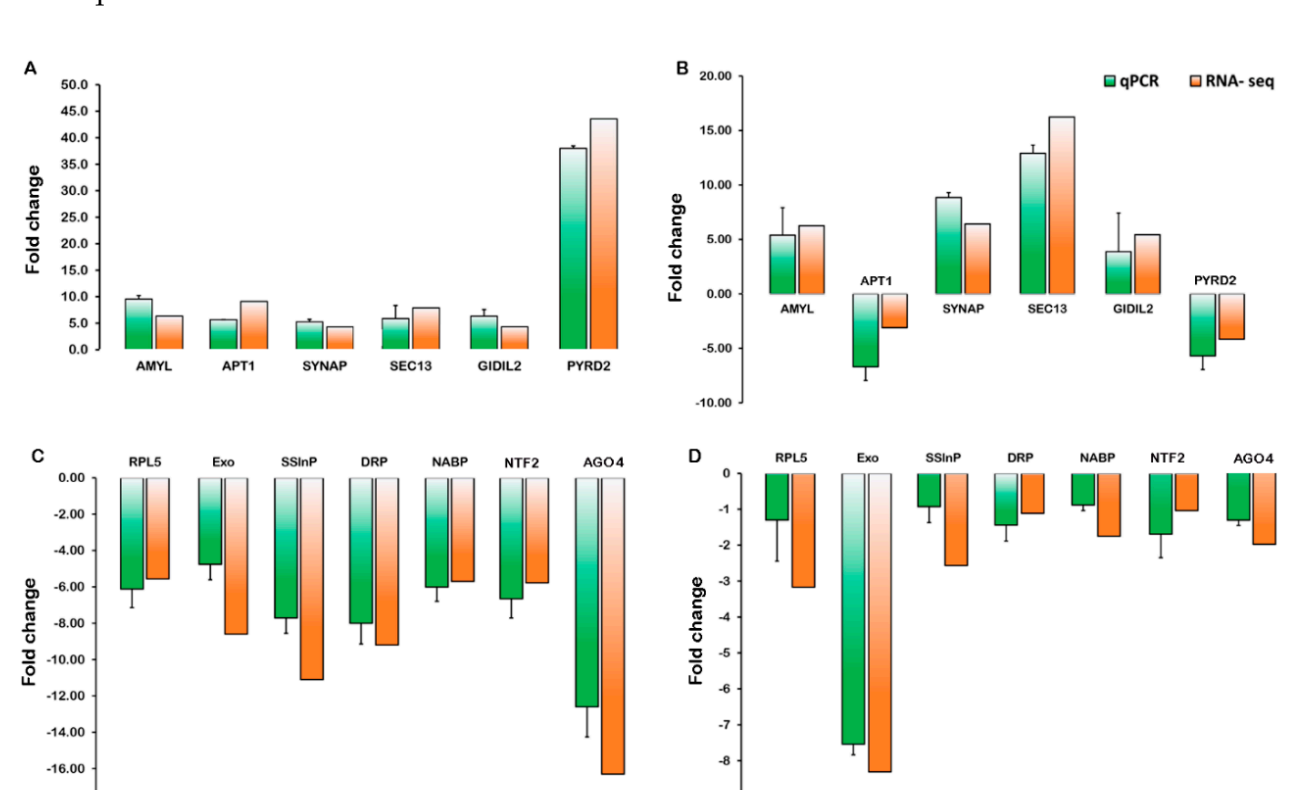
Validation of expression patterns of differentially expressed genes by RT-qPCR. Graph showing fold change of gene expression in pairwise comparison of stage 5 vs. stage 3 (**A**) and stage PT6 vs. stage 5 (**B**) of three developmental stages of control and stage 5 vs. stage 3 (**C**) and stage PT6 vs. stage 5 (**D**) of AFCVd-infected three developmental stages of male gametophyte of tobacco. AMYL: alpha-amylase; APT1: aberrant pollen transmission 1; SYNAP: synaptotagmin C2 membrane targeting protein; SEC13: protein transport SEC13-like protein; GID1L2: gibberellin receptor; PYRD2: pyruvate decarboxylase 2; RPL5: 50S Ribosomal protein L5; EXO: RNA exonuclease; SSInP: single-stranded-interacting protein 1; DRP: DNA-directed RNA polymerase; NABP: nucleic acid binding protein-K Homology domain; NTF2: nuclear transport factor 2; AGO4: argonaute 4-like protein. The qRT-PCR data (green bars) are expressed as the mean values of three experimental replicates from three independent experiments, with error bars depicting the standard error. The RNA-seq data (orange bars) are also shown for comparison.

The transcript abundance patterns of alpha-amylase (*AMYL*), aberrant pollen transmission 1 (*APT1*), synaptotagmin C2 membrane targeting protein (*SYNAP*), protein transport SEC13-like protein (*SEC13*), gibberellin receptor (*GIDIL2*), and pyruvate decarboxylase 2 (*PYRD2*) by qRT-PCR analysis showed similar patterns as the RNA-Seq data, despite some quantitative differences in expression level in CT_PT6 vs. CT_S5 (Figure 8A,B). The transcriptional levels of ribosomal protein L5 (*RPL5*), DNA-directed RNA polymerase (*DRP*), Argonaute (*AGO4*), which are primarily involved in viroid replication, were dramatically downregulated in AI_S5 compared to AI_S3 (Figure 8C). Notably, the expression of putative genes, such as single-stranded interacting protein (*SSInP*), exonuclease (*Exo*), nuclear transport factor 2 (*NTF2*), and nucleic acid binding protein (*NABP*), which assist in viroid replication showed almost similar trends of downregulation in AI_S5 and AI_PT6 (Figure 8D), which correlated with our RNA-Seq results.

## 3. Discussion

The tobacco pollen serves as an excellent model for studying various aspects of developmental physiology due to its extremely reduced but complex structure, and a synchronized series of cell divisions [26]. Pollen development is a highly coordinated and genetically programmed process involving step-by-step physiological, biochemical, and molecular changes. This study describes an integrative transcriptome-proteome approach to define the molecular changes that occur in the developing pollen under normal growth conditions and after AFCVd-infection. We also discuss the underlying molecular mechanisms and the factors that play plausible roles in the degradation and elimination of viroid in specific stages of pollen development.

### 3.1. Transcript-Protein Correlation Depicts Precise Expression Landscapes

Transcriptional profiling is only moderately accurate for predicting the changes that occur during cellular transition, since it does not account for post-transcriptional modifications and non-translated transcripts during developmental stages, which points out the importance of proteome study. Our correlation results showed that most proteins were correlated with their corresponding transcripts, but the correlation between DEPs and DEGs was modest, which could be due to post-transcriptional regulation, selective mRNA translation, mRNA storage and protein modifications within the individual developmental stages [27]. The identification of proteins related to translation (ribosomal proteins, initiation factors), maintenance, repair (heat shock proteins), and degradation (proteasome subunits) provided evidence for post-transcriptional regulatory mechanisms during developmental stages. It is worth noting that the correlation between transcripts and proteins may vary depending on several factors, in particular the stage of the tissue sample, low detection of proteins due to high hydrophobicity and excessive number of transmembrane domains [28]. Based on several previous reports [29,30,31], these factors cannot be ruled out in our study in the context of low correlation between the transcriptome and proteome datasets. In the following sections, based on the cor-DEGs/DEPs, we discussed the molecular characteristics of the pollen developmental process and the most interesting pathways affected due to AFCVd-infection during the male gametophyte development along with molecular mechanism underlying the viroid degradation and elimination in developing pollen.

### 3.2. Pollen Development Involves a Dynamic Plethora of Transcriptome and Proteome Alterations

Pollen development and the initiation of pollen tube growth has been previously reported to be accompanied by the activation of several genes with unique expression change and modulation as per the requirement of the development stage [32]. In this study, we observed a unique pattern of quantitative expression, where the number of transcripts gradually decreased during the progressive developmental stages of pollen, whereas, more number of proteins were detected in the mature PT6 stage as compared to the earlier S3 and S5 stages in both healthy and AFCVd-infected pollen (Table 2). In compliance with the above, previous studies have shown that in several instances particularly those transcripts which are involved in carbohydrate metabolism accumulate during early stages of pollen development and specifically translate during the mature stages or upon germination [33,34]. Some researchers have stipulated this phenomenon as a strategy to store reserve nutrients to be eventually used later during pollen tube germination [35]. The most dynamic regulatory trend of the nuclear-binding proteins was identified throughout the developmental stages. Interestingly, we observed that the RNA-binding proteins, Nucleolar protein 5 “ribonucleoprotein” and heterogeneous nuclear ribonucleoprotein H1 (RNP-1) which are responsible for pre-mRNA processing and m-RNA transport were abundant in S3 but depleted in the PT6 stage. However, a few of these proteins, such as the NAD-dependent RNA-binding protein-like 1, ATP-dependent RNA helicase and asparaginyl-tRNA synthetase were abundant in the PT6 stage. This indicates that some of the above factors are fundamental for active transcription and the building of the translation apparatus in the early stages of pollen development and once the basic machinery is set up, these proteins become auxiliary. In accordance with a few previous reports, we observed a marked decline in the cytosolic ribosomal proteins, and the ribosome subunits in late pollen development, demonstrating their active involvement at the initial stages, and lower persistence during the remaining development phases [22,32]. The fate of proteins during pollen growth and germination is determined by their proteasomal degradation of substrate proteins labeled by the poly-ubiquitin chain. The cor- DEGs/DEPs in CT_S5 vs. CT_S3 comparison showed the modulation of several phosphoproteins associated with protein synthesis, transport, storage, and signaling. We identified several protein kinases and kinase-interacting proteins, suggesting that protein phosphorylation is a vital process for pollen germination. The Ca^2+^/calmodulin-dependent protein kinases, cell division protein kinases and mitogen-activated protein kinases were also highly modulated, which could be important factors regulating anther development [36,37]. There were abundant cor-DEGs/DEPs identified for cell wall maintenance and cell division-related functions, such as actin cytoskeleton-regulatory complex PAN1-like protein, tubulin beta-1 chain and cell-cycle proteins, modulated in S5 and PT6 stages. The heat-shock proteins (HSPs) are synthesized inside the cell to protect the developing pollen under heat stress to allow normal pollen tube growth and germination [38]. Interestingly, the HSPs such as copper chaperone SCO1, chaperone DnaJ, and cytochrome-c oxidase copper chaperone family protein were downregulated in PT6 stage, which could be attributed to their involvement in protecting pollen particularly during the early stages and facilitating the functionality of developmental process [39,40]. On the contrary, the late embryogenesis abundant protein (LEA), possibly involved in heat-defense response, was found to be highly upregulated in the S5 stage implying its requirement in the successive stages of pollen development. The upregulation of the transmembrane proteins, exocyst complex protein and calcium-transporting ATPase1 in the initial stages of development (S3 to S5), suggesting their imperative role in the transportation of cellular components during early pollen growth. Nevertheless, the potassium chloride transporter and sodium-potassium-chloride co-transporter superfamily were upregulated in the later stages of pollen (S5 to PT6), suggesting their importance during the pollen germination and maturation stage. Conversely, some transport proteins, such as the Ras GTPase-activating binding protein, and nuclear transport factor 2, were downregulated during the transition from S5 to PT6 indicative of their non-imperative role in germinating pollen. The components of the metabolic pathways including long-chain fatty acid CoA ligase 3, glucose-6-phosphate isomerase 1, and citrate synthase were abundant in the S5 stage compared to PT6 stage of the pollen. These enzymes play crucial roles in catalyzing the formation of the intermediate primary metabolites and maintenance of carbon flux, which is required for energy supply for the homeostasis of proteins and membrane lipids [41].

### 3.3. AFCVd Infected Pollen Exhibit Several Changes in Expression of Genes and Proteins in Stagewise Comparisons

The pairwise comparisons of AFCVd-infected pollen of three stages on two levels revealed that the correlation between transcriptomic and proteomic data is not trivial. The changes at the transcriptome level were more pronounced than those at the proteome level, suggesting that AFCVd-infection leads to the intense reprogramming of transcriptome, which are reflected at the low level of proteins due to post-transcriptional modifications and other factors as described above. Moreover, in the two comparison groups, AI_PT6 vs. AI_S5 had meager number of cor-DEGs/DEPs than AI_S5 vs. AI_S3, probably because AFCVd-infection might have caused intensive degradation of transcriptome during the successive stages of pollen development, leading to a lower detection of host factors in the mature and germinating pollen.

An important feature of the plant defense response to pathogen attack is activation of various membrane-associated receptor-like kinases, which is accompanied with activation of mitogen-activated protein kinases (MAPKs) and changes in expression of immune-related genes, transcription factors, enzymes, hormones, peptides and antimicrobial chemicals [42]. The transcriptomic data showed that a large number of kinases, including adenylate kinase, MAPKs, MAP-like protein kinase, and kinase-interacting protein 1 were modulated in AI_S5 vs. AI_S3 comparison, whereas these kinases were not found to be modulated in AI_PT6 vs. AI_S5, supporting our previous speculation that activation of kinases is not obligatory due to viroid elimination in later stage of pollen development [17]. The activation of some calcium-dependent protein kinases (CDPKs) was also observed in AI_S5 vs. AI_S3 comparison. CDPKs have emerged as important Ca^2+^ sensor proteins, which function in multiple plant signal transduction pathways. Phenylalanine ammonia-lyase (PAL), a key enzyme in pathogen defense is phosphorylated by CDPK1. The enhanced expression of CDPKs suggest that they play significant roles in activation of signal transduction pathways in AFCVd-infected pollen. Auxin and brassinosteroid (BR) are two major classes of hormone, which are considered as master regulators in different plant development processes and response to various biotic and abiotic stresses [43,44]. In addition, the role of auxin and BR and their binding proteins is well-known in anther development, pollen tube growth, and subsequent fertilization [45]. There was a significant difference in DEGs related to auxins, BR and abscisic acid metabolism, which corroborates with previous findings, stating that plants employ the iterative trade-off to modulate differential organ growth upon the elicitation of immune response [46]. We observed an enhanced expression of cor-DEGs/DEPs belonging to lipid biosynthesis, tricarboxylic acid (TCA)cycle, and secondary metabolite pathways, indicative of maintaining carbon assimilation during steady development phases during viroid infection. It is reported that tissues containing the citrus exocortis viroid (CEV) reportedly exhibit altered cell wall composition and structure [47]. Consistent with this finding, our datasets revealed changes in the expression of several cell-wall related genes, which indicates that AFCVd possibly disrupts the cell wall structure of pollen during infection. Strikingly, there was a significant modulation in the expression of HSPs, proteolysis and protein degradation/modification components in AFCVd-infected pollen. Emerging reports suggest that HSPs participate in anther development and pollen-tube growth [48]. The enrichment of HSPs are indicative of their potential involvement in maintenance of pollen development in viroid pathogenesis. Another important level of regulation during biotic stress in plants is held by the histone proteins. The histones H2, H2A, and H3 were predominantly downregulated in AI_S5 vs. AI_S3, which suggests that nucleic acid metabolism is influenced by the modulation of histones during the progression of AFCVd-infection.

The integrity of pollen membrane is crucial for proper hydration and imbibition, pertaining to the germination and formation of pollen tube [35]. Sugars, such as galactose and sucrose, protect the developing pollen and provide the energy resources; the switching expression of sugar synthases and sugar transporter genes, such as inositol transporters, glucose transporters, and UDP-galactose transporters between stages indicate that they tip the scales in favor of the pollen development, by rendering energy resources as an additional layer of defense against AFCVd-infection. The co-sedimentation studies of citrus exocortis viroid (CEVd) derived small RNAs with tomato ribosomes in vivo unveiled the drastic changes in global polysome profiles, particularly in the 40S ribosomal subunit accumulation [49]. Additionally, CEVd infection induced changes in the abundance of ribosomal proteins and 18S rRNA maturation process, thereby regulating efficiency of translation. In this study, we found that AFCVd-infection triggers the downregulation of genes involved in ribosome biogenesis. The ribosomal stress response, accompanied with perturbation of translational efficiency, low concordance of transcripts and proteome in AFCVd-infected pollen (compared to the control pollen) could be explained by differences in protein turnover rates due to viroid infection.

In addition to the above, we also found the modulation of some genes, which exclusively appeared in AFCVd-infected pollen. For instance, there was a marked change in the expression pattern of the LEA protein both at the transcriptome and proteome level during AFCVd-infection. Reportedly, LEA proteins are highly accumulated in desiccation tolerant tissues like pollen, endowing them to withstand water-deficit and pathogen-stress conditions [50,51]. Our findings corroborate with the above where LEA was differentially expressed at both transcript and protein levels in AI_S5 vs. AI_S3 comparison, suggesting that LEA proteins are required for the maintenance of the pollen entity against AFCVd-infection. Some of the major transcription factor (TF) subfamilies are involved in plant responses to abiotic and biotic stress. They actively participate in the crosstalk between the host plant defense machinery and pathogen receptors to protect the host against pathogen invasion [52]. Consistent with the previous findings, we observed the upregulation of the stress-responsive genes and TFs, such as dehydration-responsive protein (DREB), ethylene-responsive factor (ERF), calmodulin, and universal stress protein (UspA) in AFCVd-infected pollen in AI_S5 and AI_S3, suggesting their potential involvement in plant defense pathways against viroids. The proper cytoskeleton organization is important for pollen tube growth and stability. The cell wall architecture is controlled by several components that drive the intracellular transport within the cell and biotic stress can result in modulation of these components [53]. There was an increase in the expression pattern of the cell division proteases, pectinases, and lipases during the transition from AI_S3 to AI_S5, suggesting that intense pressure operates during AFCVd-infection to maintain the cytoskeleton organization and cell wall architecture. Overall, our results demonstrate the dynamic nature of the pollen-AFCVd interaction, which is more pronounced at the transcriptome level than the variation at protein levels.

### 3.4. RNA Stabilization/Destabilization Proteins Participate in Viroid Eradication in Developing Pollen

In general, host-pathogen interactions are complex and dynamic phenomena that occur on different levels, where pathogen deploys diverse immune suppression tactics to colonize themselves, while the host employs counterstrategies to protect itself against the invading pathogen [54]. This congruent phenomenon was observed in our AFCVd-pollen interaction study, where viroids tend to stabilize themselves by inducing the stabilization factors, whereas host factors contribute to viroid destabilization, degradation, and elimination. The current study traced many viroid-associated stabilization/destabilization factors, which were further validated through RT-qPCR experiments. Therefore, it is tempting to speculate and discuss the plausible role of these factors. Nuclear-replicating viroids utilize DNA-dependent RNA pol II for replication, a process that involves the association of splicing variant of transcription factor IIIA (TFIIIA-7ZF) [55]. The splicing event and the expression of TFIIIA-7ZF is regulated by RPL5, which can directly interact with viroid (PSTVd). The direct interaction of viroid (PSTVd) with RPL5 for optimized expression of TFIIIA-7ZF has been shown as an integral phenomenon for modulation of viroid expression [56]. The decreasing viroid levels due to lack of replication was correlated with pronounced imbalance in the expression level of *DRP* and *RPL5* during the AI_S3 to AI_S5 transition. In addition to the role of AGO proteins in gene regulation pathway in plants, they are reported to exert the plausible influence on the viroid replication cycle [57]. Our previous study has shown that the expression of *AGO* increases during viroid infection and further decreases in the later stages of pollen development [17]. The current study also found the significant modulation of AGO protein-encoding genes including *AGO4*, indicative of their influential role in viroid propagation and degradation during the gradual process of pollen development. The interaction of elongation factor 1-alpha (eEF1A) with viroid has been previously demonstrated to be important in the replication of peach latent mosaic virus [58]. The significant downregulation eEF1A could hypothesize the biological implications of this factor in viroid elimination. In addition, we found several pollen developmental nucleases (Tudor nuclease) upregulated in AFCVd-infection, which have an established role in viroid elimination [15,17]. The exosome complex of 3′→5′ exonucleases serve as a key component of the RNA surveillance machinery and required for a quality control check for the subsequent cleavage of aberrant pre-mRNAs, pre-tRNAs, and pre-rRNAs [59]. As a protection mechanism against exonucleases, viroid has evolved the compact but imperfect secondary structures to ensure complete replication [13]. The significant downregulation of RNA exonuclease and enhancer of mRNA de-capping indicates that AFCVd could subvert their cellular level to stabilize themselves and favor their replication during pollen stage transition.

These findings indicate that the pollen interactions with AFCVd lead to the transcriptional reprogramming of many cellular and physiological processes and in this battleground the host factors overcome the viroid-induced factors favoring their stabilization, which consequently cause the elimination of viroid from developing pollen in tobacco. However, the functional significance of viroid eradication related genes identified here needs further investigation.

In conclusion, this study provides an extensive and detailed information of differentially regulated transcriptome and proteome in the developing tobacco pollen. In addition, the present study may contribute to our understanding the molecular mechanism underlying the response of pollen during the viroid infection and unique mechanism of viroid degradation and elimination in pollen.

## 4. Materials and Methods

### 4.1. Plant Materials, Viroid Infection, and Detection in Pollen

The wild type *N. tabacum* (cv. Samsun) plants were grown from seeds until at the four-leaf stage and biolistically inoculated with AFCVd infectious dimeric constructs using a Helios gene gun (Bio-Rad, CA, USA) as described previously [60] and repeated at least five times. The mock-inoculated plants were used as control. The flower buds were isolated during the flowering season from both CT and AI *N. tabacum* of stages distinguished according to their length and morphology in the flowering season of the year 2018–2019 [61]. The buds were then used to isolate the early bicellular pollen (stage 3; S3), late bicellular pollen (stage 5; S5), and the 6-hour old germinated pollen (PT6) at 4 °C. The intact anthers of CT and AI of all three stages were taken from approximately 20 buds per sample. The anthers were carefully cracked in 2 mL of 5% sucrose using pre-chilled mortar and pestle, and vortexed in a 50 mL falcon tube to release the pollen. The debris was removed by filtering the suspension through a nylon mesh. The filtrate was collected, centrifuged at 2000 g for 5 min at 4 °C and the supernatant was collected. The residual debris was removed from the pollen pellet and the isolated pollen was weighed and stored at −80 °C until further analysis (RNA and protein isolation).

To isolate germinating pollen, anthers were isolated from flowers at stage 6 (one day before anthesis) and kept in a petri dish overnight for dehiscence. The dried mature pollen was separated from the anther debris using a nylon mesh, weighed and stored at −20 °C until further analysis. Afterwards, 10 mg of mature pollen was kept at room temperature for 5 min, vortexed in 1 mL of sucrose-mineral medium buffered with MES (SMM-MES) [17,62] for 4 min, and was transferred to a conical flask to make the final volume of the pollen suspension up to 10 mL (concentration of 1 mg ml^−1^). The suspension was incubated at 27 °C for 6 h (160 rpm for the first 3 h followed by 60 rpm for the next 3 h) in a shaker incubator to cultivate the pollen. Subsequently, the pollen culture was filtered through a nylon mesh under mild pressure, weighed immediately, and stored at −80 °C until further analysis (RNA and protein isolation).

Total RNA was isolated from three developmental stages of CT and AI pollen using Concert™ reagent following manufacturers’ protocol (Plant RNA Purification Reagent, Invitrogen, Carlsbad, CA, USA). The RNA was first subject to on-column DNase digestion (RNeasy Plant Total RNA kit, Qiagen, Hilden, Germany) followed by an additional cleavage with the DNA-free™ DNA removal kit (Life Technologies, Brno, Czech Republic). The presence of viroid in the samples was confirmed by a combination of reverse transcription PCR (RT-PCR) using the AFCVd-specific primers (Table S1) and dot blot analysis as described previously [63].

### 4.2. RNA Extraction, Illumina Sequencing, and Data Processing

The DNase treated total RNA isolated from three developmental stages (S3, S5, and PT6) of CT and AI was quantified by the NanoDrop 2000 spectrophotometer (Thermo Scientific, Waltham, MA, USA) and integrity was determined in the Agilent 2100 Bioanalyzer (Agilent, CA, USA) using the RNA 6000 Nano assay kit (Agilent, CA, USA). The isolation of mRNA was performed using the PolyATtract mRNA isolation system IV (Promega Corporation, Madison, WI, USA) and used for cDNA preparation. Briefly, 1.5 μg of mRNA samples (RNA integrity number > 7.0) were used for cDNA synthesis using the Universal RiboClone cDNA synthesis system (Promega Corporation, Madison, WI, USA) following the manufacturer’s instructions. The cDNA (500–600 ng) was nebulized to shear it into smaller fragments and those ranging from 300–800 bp were used for cDNA library preparation employing the TruSeq RNA Library Preparation Kit. The quality of libraries was examined using the Agilent 2100 High Sensitivity DNA kit (Agilent Technologies, Santa Clara, CA, USA). The high-quality constructed libraries were subject to paired-end sequencing on the Illumina HiSeq 2500 platform (Illumina, San Diego, CA, USA). The obtained raw sequencing data are deposited in the Sequence Read Archive (SRA) submission of National Centre for Biotechnology (NCBI) are available under the submission numbers: PRJNA673361 (CT_S3), PRJNA669576 (CT_S5), PRJNA673392 (CT_PT6), PRJNA673793 (AI_S3), PRJNA673782 (AI_S5) and PRJNA673392 (AI_PT6). The raw sequencing data were subjected to quality trimming, removal of adapter, low quality reads, k-mers contamination using the fastp software [64]. The quality of reads was assessed based on their error rate, GC-contents, assigned Phred score of Q20, Q30 using the FASTQC toolkit [65], and read lengths ≥ 45 bp on both sides of the paired-end format were selected for downstream analysis. The clean reads derived from three developmental stages of pollen used to construct separate reference-based transcript assemblies and mapped and aligned to the transcriptome sequence of *N. tabacum* (ftp://ftp.solgenomics.net/genomes/Nicotiana_tabacum/edwards_et_al_2017/assembly/) using StringTie software [66]. The reads containing discordant alignments were rejected and uniquely localized reads were used to calculate the read counts for each gene. The fragments per kilobase of transcript per million mapped reads (FPKM value) for each sample was determined by the expectation-maximization (RSEM) [67]. The differential gene expression analysis was performed using the Bioconductor software package DESeq2 [68] with the default parameters. False discovery rate (FDR) of 0.05 and log ratio greater than 1 (two-fold change) between the samples was used to determine the significant differentially expressed genes (DEGs). A principal component analysis (PCA) was performed to explore the relationships in gene expression among the CT and AI samples using the vegan package in R (v. 3.1.2).

### 4.3. Protein Isolation and LC-MS/MS Analysis

Total protein was isolated from pollen samples of three developmental stages (S3, S5, and PT6) of CT and AI using the TRI Reagent solution (Sigma-Aldrich, St. Louis, MO, USA) following the standard protocol [33]. All isolated protein samples were processed using filter-aided sample preparation (FASP) protocol [69,70], using Microcon 30-kDa filter unit (Millipore; MILLMRCF0R030, Darmstadt, Germany), including alkylation step (iodoacetamide). Trypsin (sequencing grade; Promega, Madison, WI, US) was used for protein digestion (overnight at 37 °C). The resulting tryptic peptides mixture was cleaned by liquid–liquid extraction using ethyl acetate (3 iterations) [71]. The peptide mixtures were subject to LC-MS/MS employing the RSLCnano system connected to the Orbitrap Q Exactive HF-X spectrometer (Thermo Fisher Scientific, Prague, Czech Republic). The digested peptides were online desalted on a trapping column (Acclaim™ PepMap™ 100 C18, dimensions 300 μm × 5 mm, 5 μm particles; part number 160454), concentrated, and eluted at a flow rate of 300 nl min^−1^ onto an analytical column (Acclaim™ PepMap™100 C18, 3 μm, 75 μm × 500 mm; Thermo Fisher Scientific, Prague, Czech Republic) by the 130 min gradient program (2–35% of mobile phase B; A: 0.1% FA in water; B: 0.1% FA in 80% ACN). Digital PicoView 550 (New Objective) ion source with Active Background Ion Reduction Device, ESI Source Solutions (ABIRD) was used. MS data were acquired in a data-dependent strategy (top20; survey scan 350–2000 m/z, resolution 120,000, target value 3 × 10^6^, maximum injection time 50 ms). HCD MS/MS (27% relative fragmentation energy) spectra were acquired (target value 10,000, resolution 15,000, maximum injection time 50 ms, isolation window 1.2 m/z) with dynamic exclusion enabled for 40 s after one MS/MS spectra acquisition.

The analysis of the mass spectrometric RAW data files was carried out using the MaxQuant v1.6.2.10 software using default settings. MS/MS ion searches were done against modified Common Repository of Adventitious Proteins (cRAP) database based on The Global Proteome Machine database, and tobacco protein database (Sol Genomics Network); version from 13.4.2017, number of protein sequences 69,500. Oxidation of methionine and proline, deamidation (N, Q) and acetylation (protein N-terminus) as optional modification, carbamidomethylation (C) as fixed modification. Match between runs was set among all analyzed samples. Protein intensities reported in proteinGroups.txt were further evaluated using the software container environment (GitHub; version 3.7.2a).

Sequence comparisons were carried out with DNASIS v2.6 (Hitachi Software Engineering Company, Tokyo, Japan). Protein domain sequence analyses were performed using InterProScan module of Geneious Prime v2019.04. The PCA analysis of differentially expressed proteins at three different stages of pollen development of CT and AI samples was conducted using the “vegan” package in R version 3.1.2.

### 4.4. Correlation Analyses of Transcriptome and Proteome Profiles and Functional Analysis

The correlation between gene and corresponding protein were estimated based on their expression at the compared stages. Moreover, based on the significant differences in expression pattern of genes and corresponding proteins between the compared stages, they were designated as correlated differentially expressed correlated genes and proteins (cor-DEGs/DEPs). All the cor-DEGs/DEPs were annotated according to gene ontology (GO) terms using the Blast2GO program describing three functional groups: biological process, molecular function, and cellular component. The GO enrichment analysis of the cor-DEGs/DEPs was performed using the Gene Set Enrichment Analysis (GSEA) of clusterProfiler Bioconductor package [72]. A *p*-value < 0.05 was used as the threshold to determine the significant enrichments of GO terms. The protein pathway analysis was performed by mapping the cor-DEGs/DEPs sequence against Kyoto Encyclopedia of Genes and Genome (KEGG) database using the online KEGG Automatic Annotation Server (KAAS) (http://www.genome.jp/kegg/kaas/). The web based WebGestalt tool (http://www.webgestalt.org) was used to perform the KEGG pathway enrichment analysis [73] and the top results with the false discovery rate (FDR) adjusted *p*-value < 0.05 were considered significant. Furthermore, MapMan software [74] was utilized to generate graphical overview of the biological, metabolic and regulatory pathways for the cor-DEGs/DEPs. To gain insights into the developmental and conditional defense behavior nice visualization of expression profiles at transcriptome and proteome level, the clustering analysis was performed by GeneCluster 2.0 software [75]. For clustering analysis, the FPKM values and LFQ intensities of biological replicates for all detected genes and protein groups were averaged, whereby replicates with zero value was not considered.

### 4.5. Validation of cor-DEGs/DEPs by Quantitative Real-Time PCR (RT-qPCR) Analysis

To validate the results of differential expression obtained by using RNA-seq data, six cor-DEGs/DEPs that were potentially involved in the pollen development and seven cor-DEGs/DEPs that were potentially connected to viroid degradation were subjected to quantitative real time PCR. Gene specific primer pairs (Table S6) were designed using PerlPrimer software [76]. Total RNA extracted for library construction of three developmental stages (S3, S5, and PT6) of CT and AI was used for quantitative real-time PCR (RT-qPCR) analysis. After the removal of genomic DNA using DNase I (Promega, San Luis Obispo, CA, USA), each total RNA sample was used to synthesize the first strand of cDNA using Superscript^®^ III First-strand cDNA Synthesis System (Invitrogen, Carlsbad, USA), according to the manufacturer’s instructions. The RT-qPCR experiments were conducted with 200 nM of each forward and reverse gene-specific primers (Table S6) mixed with SYBR^®^ Green supermixes (Bio-Rad, Hercules, CA, USA) according to the manufacturer’s instructions on a Thermal Cycler CFX96 Real-Time System (Bio-Rad, Hercules, CA, USA). The relative expression levels of target genes were computed using the 2^−ΔΔCT^ method, with tobacco DEAD-box ATPase-RNA-helicase (DRH) gene as the reference gene [77] to normalize the variance among samples. The reactions were carried out in three complete biological and technical replicates; the results were averaged and based on that standard error bars were indicated.

## Figures and Tables

**Table 1:** Statistics of RNA-seq analysis covering three stages of pollen development: early bicellular pollen (S3), late bicellular pollen (S5) and 6h-pollen tube (PT6) of control and apple fruit crinkle viroid (AFCVd)-infected (AI) libraries and assembly for tobacco.

Sample Name	Number of Raw Reads (Million)	Number of Clean Reads (Million)	Total Bases of Raw Reads (GB)	Total Bases of Clean Reads (GB)	GC Content of Raw Reads (%)	GC Content of Clean Reads (%)	Mean Length of Raw Reads (bp)	Mean Length of Raw Reads (bp)	Number of Mapped Reads (Million)
**CT_S3**	100.90	96.65	15.23	10.87	43.05	41.75	151	112	86.69 (89.96%)
**CT_S5**	102.13	97.00	15.41	11.08	42.67	41.05	151	113	88.36 (91.09%)
**CT_PT6**	193.22	154.70	14.41	9.98	45.97	41.88	74	64	151.20 (97.30%)
**AI_S3**	65.70	60.31	9.91	6.63	40.70	38.78	151	110	50.58 (83.86%)
**AI_S5**	119.96	115.24	18.11	13.56	44.14	42.62	151	118	107.37 (93.17%)
**AI_PT6**	229.70	203.75	17.13	14.44	44.25	42.29	74	71	185.27 (90.93%)

**Table 2:** Number of total and correlated differentially expressed (DE) transcripts and proteins in S3, S5, and PT6 of control (CT)and AFCVd-infected (AI) tobacco pollen (UR, upregulated; DR, downregulated).

Single Stage Comparison	Transcripts (T)		Proteins (P)		Corresponding Number of TP Pairs	
CT_S3	64,033		5321		2660 (4.0%)	
CT_S5	60,597		5286		2639 (4.2%)	
CT_PT6	46,640		6923		3428 (6.8%)	
AI_S3	63,335		5371		2672 (4.4%)	
AI_S5	60,278		5267		2618 (4.2%)	
AI_PT6	46,461		6923		3438 (6.9%)	
**Pairwise stage comparisons**	**DE_Transcripts**		**DE_Proteins**		**DE_Correlated**	
	UR	DR	UR	DR	UR	DR
CT_S5 vs. CT_S3	5003	4304	869	1365	412	395
CT_PT6 vs. CT_S5	6309	7756	1094	1014	301	545
AI_S5 vs. AI_S3	3014	1752	829	1395	237	248
AI_PT6 vs. AI_S5	2533	3307	709	600	68	24

**Table 3:** Classification statistics for differentially expressed correlated genes [up-regulated (UR) and down-regulated genes (DR)] in three stages of pollen development: early bicellular pollen (S3), late bicellular pollen (S5), and 6h-pollen tube (PT6) of control (CT) and AFCVd-infected (AI) tobacco according to KEGG pathway analysis.

KEGG Categories	CT S5 vs. S3		CT PT6 vs. S5		AI S5 vs. S3		AI PT6 vs. S5	
Metabolism	UR	DR	UR	DR	UR	DR	UR	DR
Carbohydrate Metabolism	92	8	41	71	47	18	12	6
Energy metabolism	19	3	12	26	9	5	7	3
Lipid metabolism	29	1	16	17	14	6	2	0
Nucleotide metabolism	8	4	8	8	9	3	3	1
Amino acid metabolism	42	10	21	35	25	16	2	2
Metabolism of other amino acids	8	4	6	6	4	2	2	1
Glycan biosynthesis and metabolism	0	0	2	7	3	2	0	0
Metabolism of cofactors and vitamins	8	6	6	11	3	4	4	0
Metabolism of terpenoids and polyketides	8	2	2	3	3	1	0	0
Biosynthesis of other secondary metabolites	13	6	7	9	5	6	3	1
Xenobiotics biodegradation and metabolism	6	1	2	2	3	1	2	0
Enzyme families	0	0	0	0	0	0	0	0
**Genetic information processing**								
Transcription	0	21	1	13	0	10	2	1
Translation	5	45	8	67	2	33	8	3
Folding, sorting and degradation	14	14	12	37	5	8	4	0
Replication and repair	0	9	4	9	0	6	5	0
RNA family	0	0	0	0	0	0	0	0
**Cellular Process**								
Transport and catabolism	25	7	27	11	7	3	2	0
Cell growth and death	9	11	9	23	2	8	5	0
Cellular community - eukaryotes	3	1	8	5	2	1	0	0
Cellular community - prokaryotes	2	0	2	0	1	1	0	1
Cell motility	3	1	3	1	3	1	0	0
**Environmental information processing**								
Membrane transport	0	0	0	0	0	0	0	0
Signal transduction	44	13	56	56	17	11	5	0
Signaling molecules and interaction	0	0	0	0	0	0	0	0
**Others**	140	96	160	341	52	74	80	4
Annotated	282	255	203	380	131	171	50	16
Query dataset	412	395	301	542	237	248	67	24

## Supplementary Materials

The following are available online at https://www.mdpi.com/1422-0067/21/22/8700/s1.
